# Supplementary material for: Epigenetic Modifications of White Blood Cell DNA Caused by Transient Fetal Infection with Bovine Viral Diarrhea Virus
Source: Viruses. 2024 May 1;16(5):721. doi: 10.3390/v16050721 (PMC11125956; doi:10.3390/v16050721)
Supplement: Supplementary file 1 [file viruses-16-00721-s001.zip › Figure S1 BVDV RTPCR hvc 04202024.pdf]

A

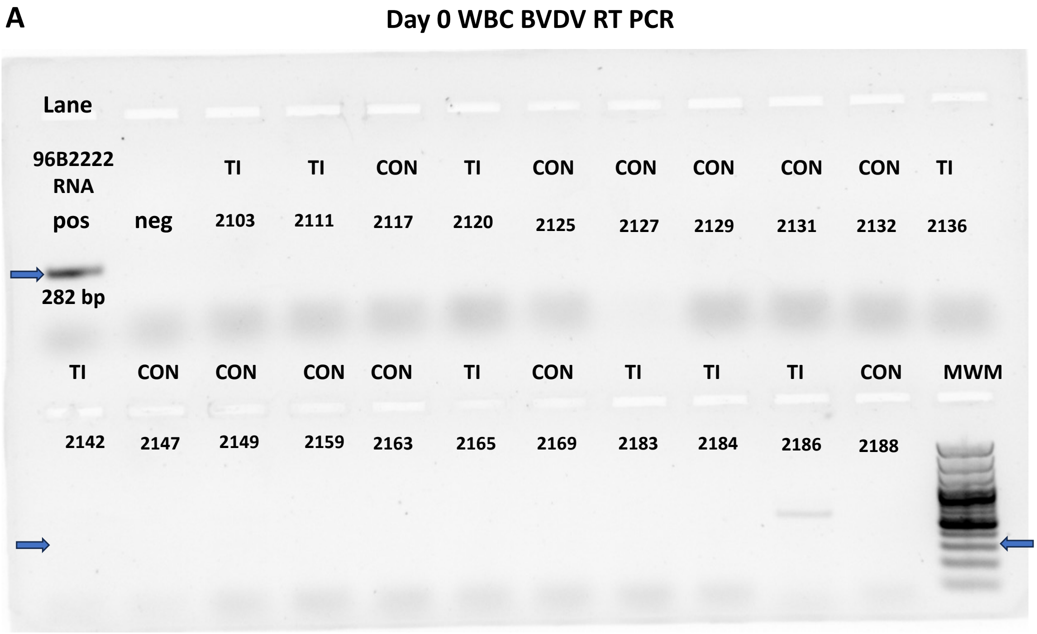

B

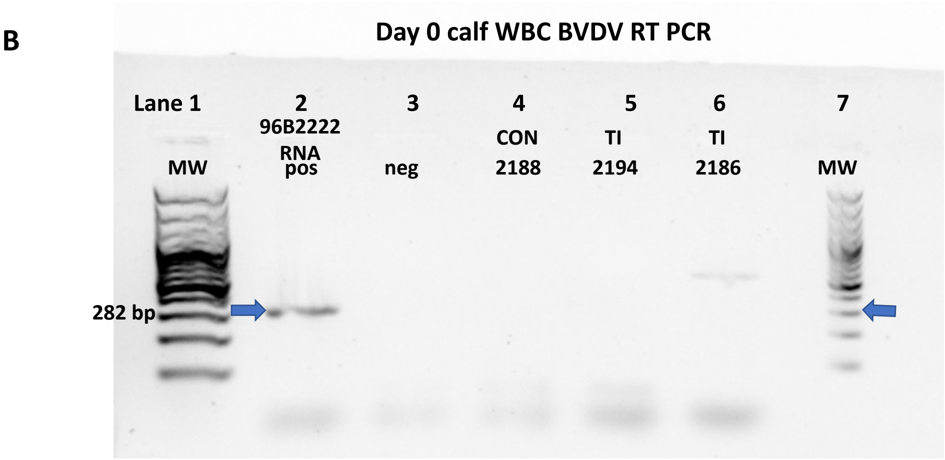

Figure S1. BVDV RT PCR analysis of Day 0 white blood cell RNA. The 9682222 BVDV RNA control amplified using specific oligonucleotide primers and migrated to the expected ~282 bp band. The expected migration of this band is noted the blue arrow. All control and TI calves were negative for BVDV RNA A & B (panels A and B). The 2186 TI calf had an odd band at a molecular weight higher than expected for BVDV RNA. For this reason, this calf white blood cell RNA was repeated for BVDV RT PCR amplification, which resulted in the same higher molecular weight band. This band clearly is not BVDV based on the expected size and must represent amplification of a non-target RNA in this calf only. This does not change interpretation that all calves were negative for BVDV on Day 0.
