# Supplementary material for: Epigenetic Modifications of White Blood Cell DNA Caused by Transient Fetal Infection with Bovine Viral Diarrhea Virus
Source: Viruses. 2024 May 1;16(5):721. doi: 10.3390/v16050721 (PMC11125956; doi:10.3390/v16050721)
Supplement: Supplementary file 1 [file viruses-16-00721-s001.zip › Figure S6 D0 KEGG Analysis of Cytokine-Cytokine Receptor Signaling.pdf]

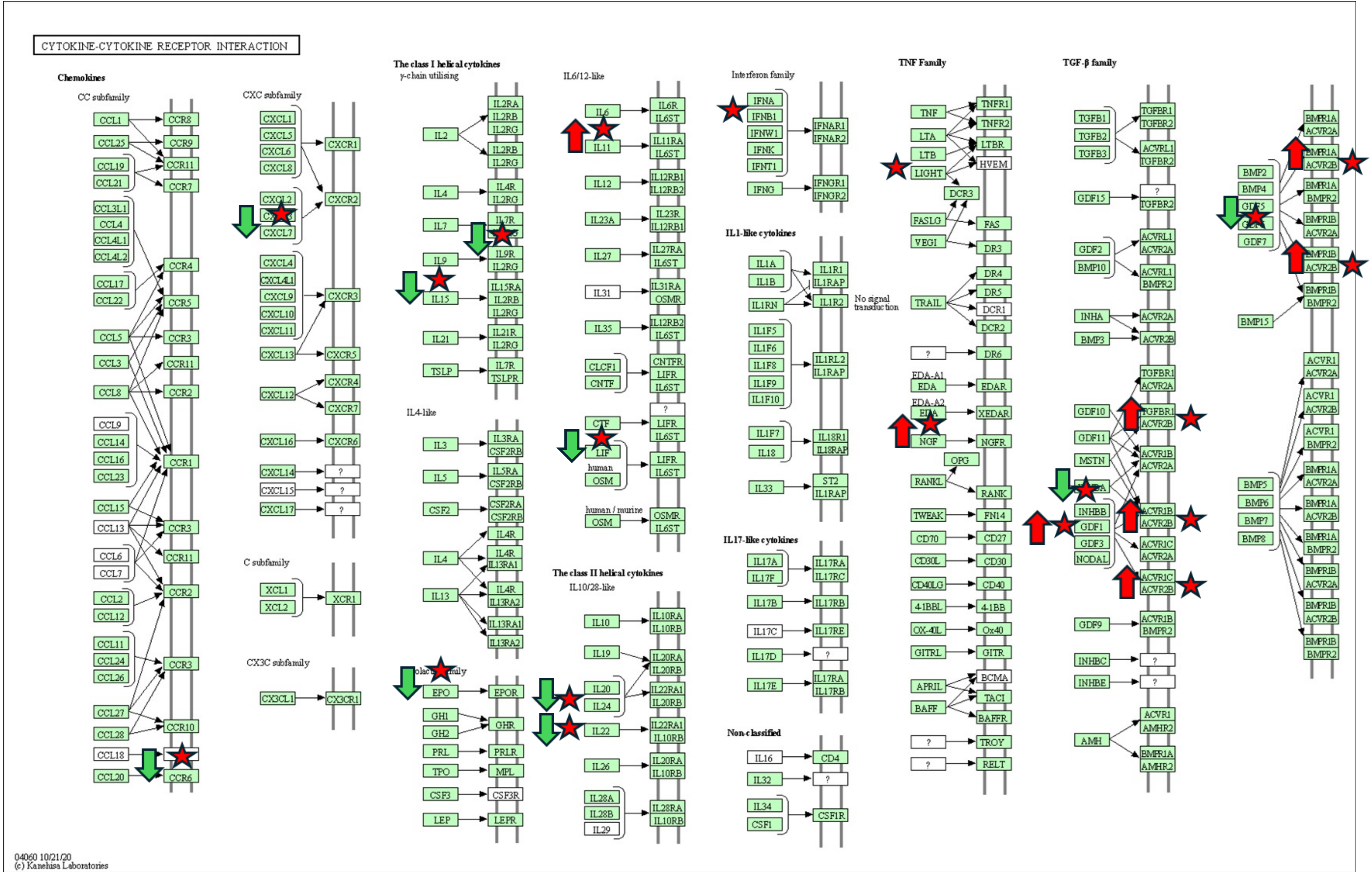

CYTOKINE-CYTOKINE RECEPTOR INTERACTION
Chemokines
CC subfamily
CCL1
CCL25
CCL19
CCL21
CCL3L1
CCL4
CCL4L1
CCL4L2
CCL17
CCL22
CCL5
CCL3
CCL8
CCL9
CCL14
CCL16
CCL23
CCL15
CCL13
CCL6
CCL7
CCL2
CCL12
CCL11
CCL24
CCL26
CCL27
CCL28
CCL18
CCL20
CCR8
CCR9
CCR11
CCR7
CCR4
CCR5
CCR3
CCR11
CCR2
CCR1
CCR3
CCR11
CCR2
CCR3
CCR10
CCR6
CXC subfamily
CXCL1
CXCL5
CXCL6
CXCL8
CXCL2
CXCL7
CXCL4
CXCL4L1
CXCL9
CXCL10
CXCL11
CXCL13
CXCL12
CXCL16
CXCL14
CXCL15
CXCL17
CXCR1
CXCR2
CXCR3
CXCR5
CXCR4
CXCR7
CXCR6
?
?
?
C subfamily
XCL1
XCL2
XCR1
CX3C subfamily
CX3CL1
CX3CR1
The class I helical cytokines
γ-chain utilising
IL2
IL2RA
IL2RB
IL2RG
IL2RB
IL2RG
IL4
IL4R
IL2RG
IL7
IL7R
IL9
IL9R
IL2RG
IL15
IL15RA
IL2RB
IL2RG
IL21
IL21R
IL2RG
TSLP
IL7R
TSLPR
IL4-like
IL3
IL3RA
CSF2RB
IL5
IL5RA
CSF2RB
CSF2
CSF2RA
CSF2RB
IL4
IL4R
IL4R
IL13RA1
IL13
IL4R
IL13RA2
IL13RA1
IL13RA2
EPO
EPOR
GH1
GH2
GHR
PRL
PRLR
TPO
MPL
CSF3
CSF3R
LEP
LEPR
IL6/12-like
IL6
IL6R
IL6ST
IL11
IL11RA
IL6ST
IL12
IL12RB1
IL12RB2
IL23A
IL23R
IL12RB1
IL27
IL27RA
IL6ST
IL31
IL31RA
OSMR
IL35
IL12RB2
IL6ST
CLCF1
CNTF
CNTFR
LIFR
IL6ST
?
CTF
LIFR
IL6ST
LIF
human
OSM
LIFR
IL6ST
human / murine
OSM
OSMR
IL6ST
The class II helical cytokines
IL10/28-like
IL10
IL10RA
IL10RB
IL19
IL20RA
IL20RB
IL20
IL24
IL22RA1
IL20RB
IL22
IL22RA1
IL10RB
IL26
IL20RA
IL10RB
IL28A
IL28B
IL29
IL28RA
IL10RB
Interferon family
IFNA
IFNB1
IFNW1
IFNK
IFNT1
IFNAR1
IFNAR2
IFNG
IFNGR1
IFNGR2
IL1-like cytokines
IL1A
IL1B
IL1RN
IL1R1
IL1RAP
IL1R2
No signal transduction
IL1F5
IL1F6
IL1F8
IL1F9
IL1F10
IL1RL2
IL1RAP
IL1F7
IL18
IL18R1
IL18RAP
IL33
ST2
IL1RAP
IL17-like cytokines
IL17A
IL17F
IL17RA
IL17RC
IL17B
IL17RB
IL17C
IL17RE
IL17D
?
IL17E
IL17RA
IL17RB
Non-classified
IL16
CD4
IL32
?
IL34
CSF1
CSF1R
TNF Family
TNF
LTA
LTB
LIGHT
TNFR1
TNFR2
LTBR
HVEM
DCR3
FASLG
VEGI
FAS
DR3
TRAIL
DR4
DR5
DCR1
DCR2
?
DR6
EDA-A1
EDA
EDAR
EDA-A2
XEDAR
NGF
NGFR
OPG
RANKL
RANK
TWEAK
FN14
CD70
CD27
CD30L
CD30
CD40LG
CD40
4-1BBL
4-1BB
OX-40L
Ox40
GITRL
GITR
APRIL
BAFF
BCMA
TACI
BAFFR
?
TROY
?
RELT
TGF-β family
TGFB1
TGFB2
TGFB3
TGFBR1
TGFBR2
ACVRL1
TGFBR2
GDF15
?
TGFBR2
GDF2
BMP10
ACVRL1
ACVR2A
ACVRL1
BMPR2
INHA
BMP3
ACVR2A
ACVR2B
GDF10
GDF11
MSTN
INHBB
GDF1
GDF3
NODAL
TGFBR1
ACVR2A
TGFBR1
ACVR2B
ACVR1B
ACVR2A
ACVR1B
ACVR2B
ACVR1C
ACVR2A
ACVR1C
ACVR2B
GDF9
ACVR1B
BMPR2
INHBC
?
INHBE
?
AMH
ACVR1
AMHR2
BMPR1A
AMHR2
BMP2
BMP4
GDF7
BMP15
BMPR1A
ACVR2A
BMPR1A
ACVR2B
BMPR1A
BMPR2
BMPR1B
ACVR2A
BMPR1B
ACVR2B
BMPR1B
BMPR2
ACVR1
ACVR2A
ACVR1
ACVR2B
ACVR1
BMPR2
BMP5
BMP6
BMP7
BMP8
BMPR1A
ACVR2A
BMPR1A
ACVR2B
BMPR1A
BMPR2
BMPR1B
ACVR2A
BMPR1B
ACVR2B
BMPR1B
BMPR2
04060 10/21/20
(c) Kanehisa Laboratories
