# Supplementary figures and images for: Epigenetic Modifications of White Blood Cell DNA Caused by Transient Fetal Infection with Bovine Viral Diarrhea Virus
Source: Viruses. 2024 May 1;16(5):721. doi: 10.3390/v16050721 (PMC11125956; doi:10.3390/v16050721)

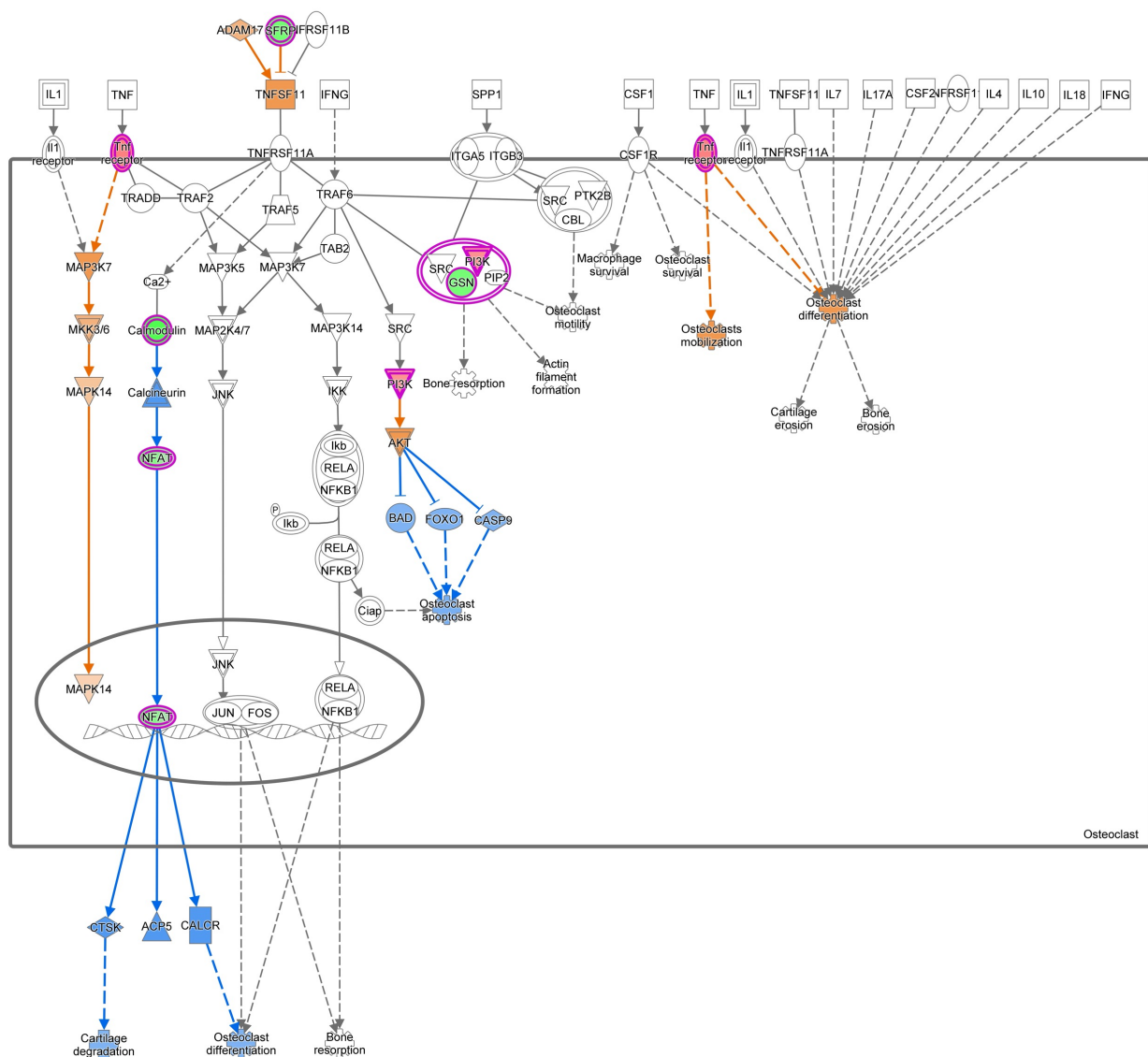

Supplement: Supplementary file 1 [file viruses-16-00721-s001.zip › Figure S4 Osteoclast.pdf]

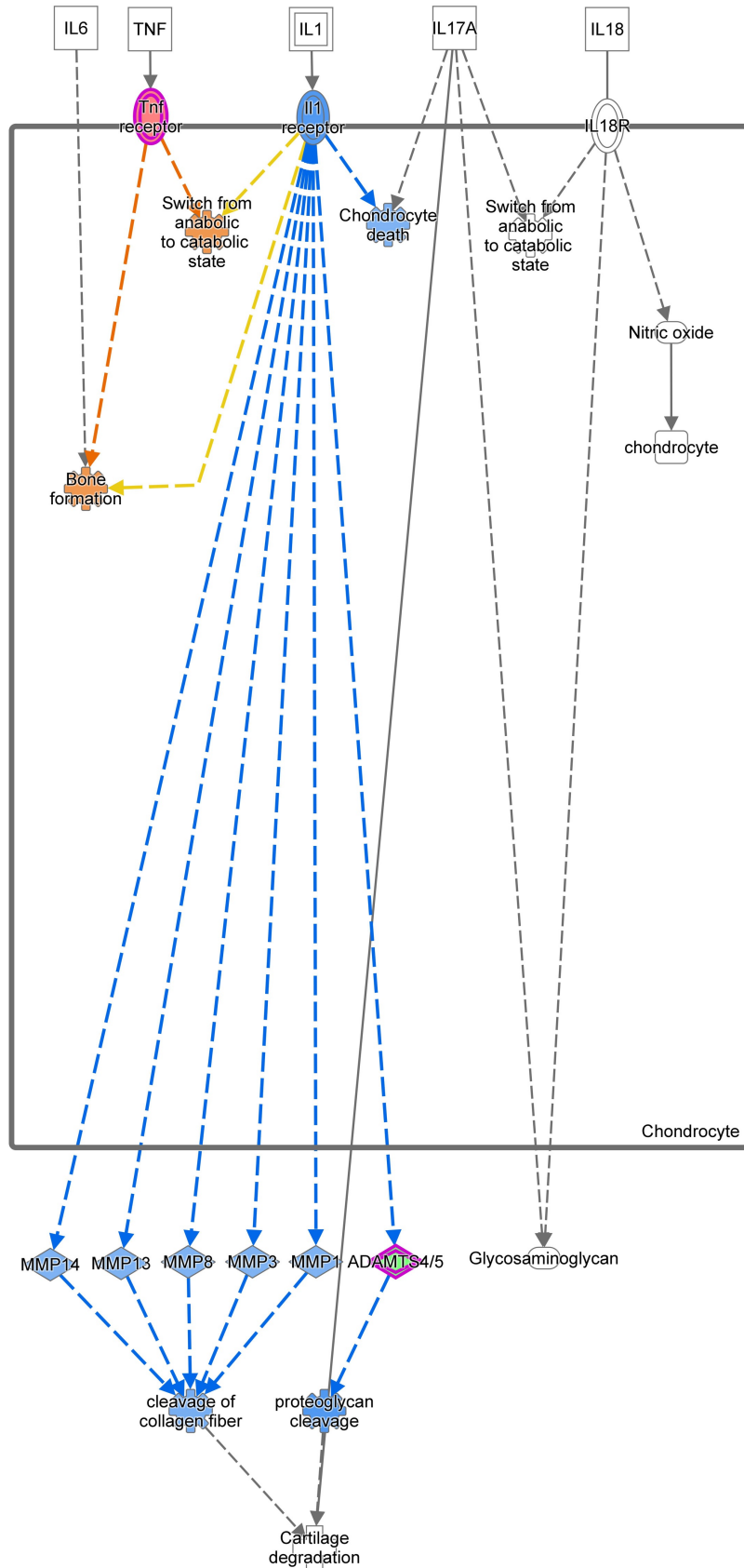

Supplement: Supplementary file 1 [file viruses-16-00721-s001.zip › Figure S5 Chondrocytes March 11.pdf]
